# C1-2 cyst presenting with syringobulbia: a case report

**DOI:** 10.1093/jscr/rjab097

**Published:** 2021-04-06

**Authors:** Michael J Yang, Knarik Arkun, James T Kryzanski

**Affiliations:** Department of Neurosurgery, Tufts Medical Center, Boston, MA 02111, USA; Department of Neurosurgery, Tufts Medical Center, Boston, MA 02111, USA; Department of Pathology and Laboratory Medicine, Tufts Medical Center, Boston, MA 02111, USA; Department of Neurosurgery, Tufts Medical Center, Boston, MA 02111, USA

**Keywords:** atlantoaxial cyst, spinal synovial cyst, spinal ganglion cyst, far-lateral transcondylar approach, syringobulbia

## Abstract

Extradural atlantoaxial cysts are typically related to C1-2 degeneration. Intradural cysts may cause secondary syringobulbia depending on the size and cerebrospinal fluid flow obstruction. However, medullary syrinxes have not been previously described with extradural cysts. Treatment of symptomatic lesions involves surgical resection, often via a far-lateral approach, with consideration of fusion if C1-2 instability is present. We present a case of an extradural C1-2 cyst with intradural extension causing syringobulbia. Effective surgical resection was accomplished via a far-lateral, partial transcondylar approach without fusion. It is important to recognize that cysts of extradural origin may exhibit intradural extension and compress critical neurovascular structures.

## INTRODUCTION

Atlantoaxial cystic lesions of intradural origin include neurenteric and arachnoid cysts; extradural cysts are often associated with C1-2 degenerative pathology and include synovial- or ganglion-type cysts [1, 2]. Syringobulbia has been described with intradural cysts but not with extradural C1-2 degenerative cysts. Here, we present a unique case of a degenerative C1-2 cyst with intradural extension causing a medullary syrinx.

## MATERIALS AND METHODS

**Figure 1:**
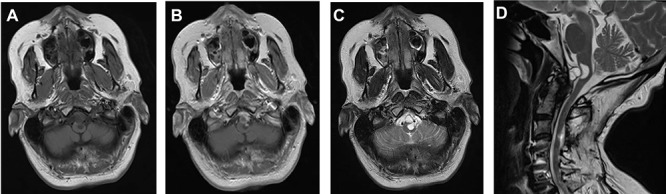
MRI findings of a ventral atlantoaxial degenerative cyst with an associated medullary syrinx; axial T1-weighted pre-contrast (**A**), axial T1-weighted post-contrast (**B**), axial T2-weighted (**C**) and sagittal T2-weighted (**D**) images demonstrate an approximately 1 × 1 cm T1-isointense, T2-hyperintense, rim-enhancing cyst at C1-2 compressing the ventral medulla with an associated a fluid-filled medullary syrinx.

**Figure 2:**
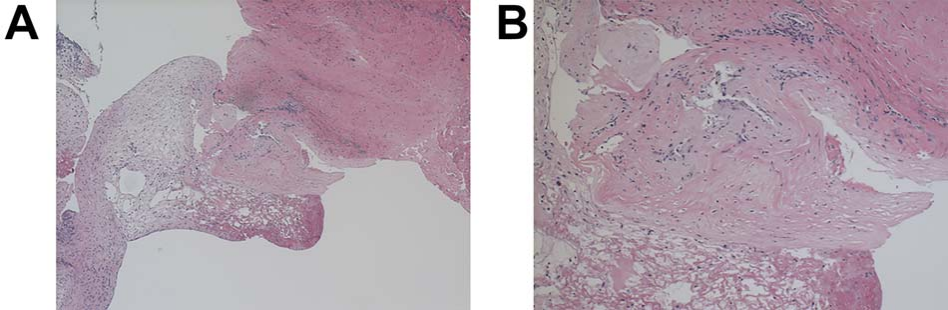
Light microscopy of the resected C1-2 cyst under ×40 (**A**) and ×100 (**B**) magnification; hematoxylin- and eosin-stained sections of cyst wall show benign fibroconnective tissue with degeneration but without epithelium or focal endothelial proliferation; GFAP, EMA, S-100, progesterone, CEA-M immunostains were negative.

**Figure 3:**
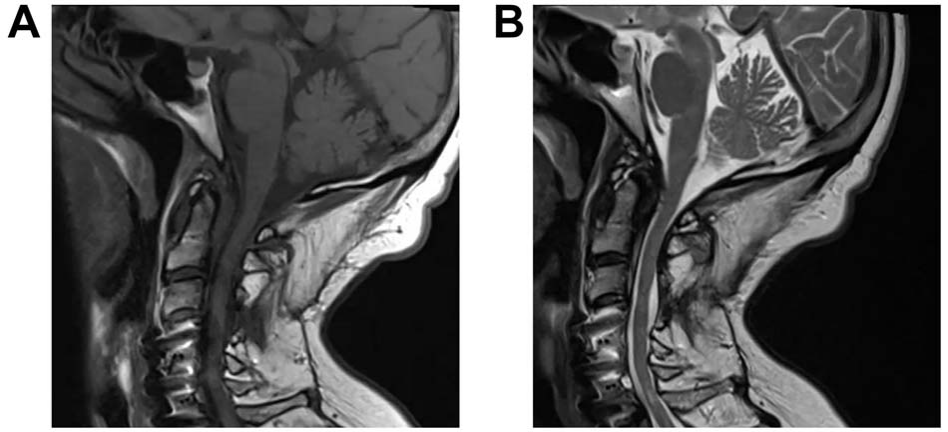
Repeat MRI 4 months post-minimal access far-lateral transcondylar approach for resection of the C1-2 degenerative cyst; sagittal T1-weighted (**A**) and T2-weighted (**B**) sequences demonstrate gross total resection of the cyst with complete resolution of the medullary syrinx.

A 64-year-old female status post-two prior spine surgeries presented with recurrent cervical myelopathy. Magnetic resonance imaging (MRI) revealed a 1-cm ventral medullary cyst (Fig. 1A–D), which had enlarged compared with prior imaging. There was significant C1-2 joint arthropathy but no overt instability. She underwent uncomplicated left minimal access far-lateral transcondylar craniotomy for cyst resection. The cyst contained viscous material and was sharply resected, with the exception of the dorsal wall densely adherent to the brainstem. Pathology specimens revealed benign fibroconnective tissue with fibrinous degeneration and focal vascular proliferation (Fig. 2A and B). Immunohistochemical staining for S-100, glial fibrillary acidic protein (GFAP), progesterone receptor (PR), epithelial membrane antigen (EMA) and carcinoembryonic antigen (CEA) was negative; these findings were consistent with a ganglion cyst. Her pain resolved and strength and balance improved post-operatively. Repeat imaging 4 months post-operation demonstrated gross total cyst resection with resolution of the bulbar syrinx and reconstitution of appropriate ventral medullary anatomy (Fig. 3A and B).

## DISCUSSION

Many types of intradural cysts have been described at the foramen magnum and atlantoaxial joint. Although rare—only 0.01% of central nervous system tumors—neurenteric cysts are among the best studied [3]. They are commonly located at the cervicothoracic junction and are associated with bony malformations, including scoliosis and spina bifida; when intracranial, they often appear in the posterior fossa [3]. Neurenteric cysts are postulated to derive from a persistent accessory canal that forms secondary to aberrant duplication of the neurenteric canal during caudal regression of the primitive streak [4]. Neurenteric cysts are ciliated columnar epithelium, recapitulating respiratory or gastrointestinal epithelium. They therefore reliably stain positive for epithelial membrane antigen (EMA), cytokeratin, carcinoembryonic antigen (CEA) and CA 19-9 but negative for glial fibrillary acidic protein (GFAP) and S-100 [3].

Arachnoid cysts are also found at the foramen magnum, although they typically have a dorsal as opposed to ventral location [5]. Arachnoid cysts have a classic and diagnostic appearance radiographically: they are discrete hypodense lesions on computed tomography (CT) and T2-hyperintense lesions on MRI following the intensity of cerebrospinal fluid (CSF). Histologically, they have delicate fibrous membranes of arachnoid cells often with meningothelial cell hyperplasia, which can be confirmed by EMA and PR immunostains.

Extradural cysts at the atlantoaxial joint include synovial and ganglion cysts, which are typically degenerative. They are uncommon, with approximately 70 cases reported [6, 7]. Degenerative spine cysts characteristically appear as T1-hypointense and T2-hyerintense lesions on MRI frequently with cyst wall enhancement [7]. At the atlantoaxial joint, these cysts are thought to form secondary to trauma to or instability in the joint itself [1, 7]. Some authors have theorized that fusion across the joint—with or without cyst resection—is necessary to relieve symptoms and prevent cyst recurrence [7]. However, cyst resection alone is sufficient for symptomatic relief in nearly 90% patients [1]. Ganglion and synovial cysts are difficult to differentiate, and the distinction is often of little clinical relevance.

Syringobulbia, when associated with upper cervical cysts, has been described exclusively with intradural pathology. Its pathophysiology is poorly understood, but most theories implicate aberrant CSF flow dynamics [8]. In our case, however, we hypothesize an unusual mechanism for syrinx formation: given the dorsal aspect of the cyst was densely adherent to the ventral medulla, the syrinx likely arose directly from cyst fluid passing into the brainstem and not indirectly from CSF flow perturbation.

The far-lateral transcondylar approach is particularly useful to treat ventral foramen magnum pathology, given its versatility and minimal brainstem retraction [9, 10]. The senior author (J.T.K.) has previously described a minimal access far lateral approach using a small paramedian incision with muscle splitting technique [9]. In our case, this approach led to successful resection of the C1-2 cyst and syrinx resolution.

In conclusion, atlantoaxial degenerative synovial cysts are primarily extradural and have management considerations different from the intradural cystic lesions in the same region. Extradural C1-2 cysts arise when there is significant arthropathy and/or instability. The literature is divided on the treatment of cysts by resection alone, fusion alone or both. Nonetheless, joint instability must be evaluated as part of the treatment algorithm. In this case, a degenerative atlantoaxial extradural cyst exhibited intradural extension with syringobulbia, a presentation heretofore seen only with intradural cysts. It is important to recognize this pathology so that concomitant C1-2 arthropathy and potential instability are addressed as part of the treatment strategy.

## CONFLICT OF INTEREST STATEMENT

None declared.
